# The CroCo cross-link converter: a user-centred tool to convert results from cross-linking mass spectrometry experiments

**DOI:** 10.1093/bioinformatics/btz732

**Published:** 2019-09-28

**Authors:** Julian Bender, Carla Schmidt

**Affiliations:** Martin Luther University Halle-Wittenberg, Institute for Biochemistry and Biotechnology, Interdisciplinary Research Center HALOmem, Charles Tanford Protein Center, 06120 Halle (Saale), Germany

## Abstract

**Motivation:**

A variety of search engines exists for the identification of peptide spectrum matches after cross-linking mass spectrometry experiments. The resulting diversity in output formats complicates data validation and visualization as well as exchange with collaborators, particularly from other research areas.

**Results:**

Here, we present CroCo, a user-friendly standalone executable to convert cross-linking results to a comprehensive spreadsheet format. Using this format, CroCo can be employed to generate input files for a selection of the commonly utilized validation and visualization tools.

**Availability and implementation:**

The source-code is freely available under a GNU general public license at https://github.com/cschmidtlab/croco. The standalone executable is available and documented at https://cschmidtlab.github.io/CroCo.

**Supplementary information:**

Supplementary data are available at *Bioinformatics* online.

## 1 Introduction

Chemical cross-linking and mass spectrometry (XL-MS) are often combined to gain low-resolution structural information on protein–protein interactions (Rappsilber, 2011; Sinz, 2014). For this, a protein or protein complex is treated with a chemical cross-linker that covalently links amino acid residues in close proximity. The proteins are then enzymatically hydrolyzed and cross-linked di-peptides are identified by MS.

As the combination of potential covalently linked peptide sequences significantly increases the database search space, specialized software tools have been developed for the analysis of XL-MS experiments. The most commonly used tools for identification of covalently linked di-peptides include xQuest (Rinner *et al.*, 2008), StavroX (Gotze *et al.*, 2012), pLink (Yang *et al.*, 2012), Kojak (Hoopmann *et al.*, 2015) and XiSearch (Mendes *et al.*, 2018). After identification of cross-linked residues, spectral annotation can manually be validated using tools such as pLabel (Li *et al.*, 2005) or xiSPEC (Kolbowski *et al.*, 2018). Identified cross-links can further be explored by mapping them on three-dimensional structures using xWalk (Kahraman *et al.*, 2011) or XlinkAnalyzer (Kosinski *et al.*, 2015) or by generating network plots using xiNet (Combe *et al.*, 2015) or xVis (Grimm *et al.*, 2015). DynamXL integrates conformational ensembles of proteins with cross-linking (Degiacomi *et al.*, 2017).

However, each software tool requires specific input formats and, therefore, most laboratories developed an individual data processing pipeline. Community standards are consequently missing (Iacobucci *et al.*, 2019). Here, we introduce CroCo, a software tool to convert results of the most commonly used cross-link search engines to a common text format that simplifies data handling and management. The text file can then be converted to input files for the post-processing tools described.

## 2 Implementation

**Fig. 1.**
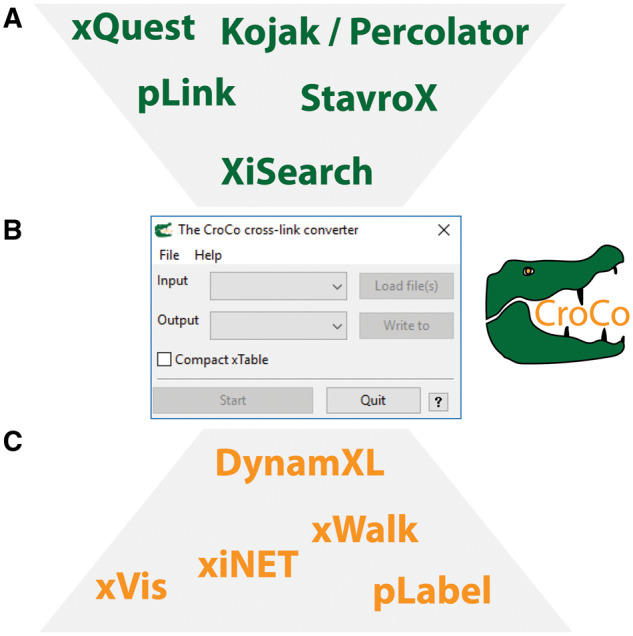
CroCo workflow. The user can select one of the available input formats (**A**) in the graphical user-interface (**B**). After selecting one of the post-processing tools compatible with CroCo (**C**) and providing the respective input and output paths, conversion can be started. If additional information is required for conversion (e.g. additional input files), CroCo will ask the user in a pop-up window

CroCo is written in Python 3.6 and relies on the pandas library for handling data tables. The graphical user-interface (GUI) is based on wxPython. CroCo is designed as a standalone executable that allows fast and easy distribution; a Python module for integration into existing workflows is also available. It is centred on collection of scripts to parse the output formats of the commonly used cross-linking search engines Kojak, StavroX, Xi, pLink and xQuest. During data conversion, the input file is internally transformed into a pandas data frame object with defined column headers. The data frame can be exported in comma-separated .csv format (called xTable) to simplify manual validation of the identified cross-links as well as data filtering. As an example, using the xTable, an input file for the pLabel spectral annotation software can be created. During manual inspection using pLabel, peptide-spectrum matches of lower quality can be removed from the xTable. The reduced table containing high-confidence cross-links can then be converted to an input file for cross-link visualization tools. A list of output formats available is shown in Figure 1.

Using the standalone GUI, the user can select the appropriate input and output formats as well as the required file paths followed by data conversion. Additional information needed to generate the xTable is requested in an additional window, if necessary. As CroCo relies on the xTable intermediate data file, it can easily be extended including additional software tools while maintaining full compatibility with the already established tools and formats.

## 3 Results

To demonstrate the use of CroCo, we chemically cross-linked homomeric pyruvate kinase from rabbit. The cross-linked protein complex was analysed following established standard protocols (Haupt *et al.*, 2017). Potential cross-links were then identified employing the various search engines compatible with CroCo. Examples of the input files and converted xTable files and a description of the column headers used are presented in the Supplementary Material. Note, that CroCo will generate columns containing additional database search results present in the corresponding input file. The addition of the original search results not required to generate the xTable can optionally be turned off during data conversion. The conversion of a selected xTable to the available output formats for data validation and visualization was tested (Supplementary Material).

## 4 Conclusion

We developed CroCo, a user-friendly conversion tool for cross-linking data management. Manual inspection followed by conversion to the xTable simplifies data exchange within the cross-linking community. The generated xTable could serve as a common data structure paving the way to reach a common reporting standard. We aim to integrate a community-based standardized reporting data-format as soon as it is defined.

## Supplementary Material

btz732_Supplementary_Data
